# *In vitro* colonic fermentation of fermented Radix Astragali by *Poria cocos* and anti-hyperuricemia mechanism based on network pharmacology and experiment verification

**DOI:** 10.3389/fnut.2024.1466702

**Published:** 2024-12-09

**Authors:** Caiyun Chen, Keyu Liu, Yishu Wang, Xinru Song, Wenjing Gao, Yanlin Wang, Yuxin Chen, Ziqi An, Changting Yin, Haiyan Wang, Shaoping Wang

**Affiliations:** ^1^School of Public Health, Binzhou Medical University, Yantai, China; ^2^Office of Academic Affairs, Binzhou Medical University, Yantai, China; ^3^School of Pharmaceutical Science, Binzhou Medical University, Yantai, China

**Keywords:** Radix Astragali, colonic fermentation, *Lactobacillus acidophilus*, hyperuricemia, network pharmacology

## Abstract

**Aim:**

This research aimed to probe the effects of fecal microbiota and *Lactobacillus acidophilus* on the metabolism of Radix Astragali (RA) and *Poria cocos* solid fermenting Radix Astragali (FRA). It further explores pharmacological effects of RA, *Poria cocos*, and FRA on HUA mouse model and the mechanisms in HUA treatment.

**Methods:**

Fecal microbiota and *Lactobacillus acidophilus* were used to ferment FRA and RA in vitro to probe the impacts of microbiota on the metabolism of active compound. A HUA mouse model was used to carry out pharmacodynamic experiment of anti-hyperuricemia. Network pharmacology and molecular docking was utilized to elucidate the underlying mechanisms of RA and *Poria cocos* in the treatment of HUA.

**Results:**

The results indicated that astragaloside IV (AG IV), total saponins, and flavonoids continuously decreased in FRA and RA during 48 h fecal microbiota colonic fermentation. During *Lactobacillus acidophilus* fermentation, in FRA, the content of AG IV peaked at 12 h with a value of 1.14 ± 0.20 mg/g; total saponins and flavonoids reached the highest values of 136.34 ± 6.15 mg/g at 12 h and 6.35 ± 0.06 mg/g at 6 h; AG IV and total saponins reached the highest values 0.63 ± 0.05 mg/g and 115.12 ± 4.12 mg/g at 12 h and 24 h in RA, respectively; and total flavonoids consecutively decreased. The counts of *Lactobacillus acidophilus* increased significantly in FRA compared with RA. Pharmacodynamic outcomes revealed that FRA effectively reduced blood levels of uric acid (UA), triglycerides (TG), xanthine oxidase (XOD), alanine aminotransferase (ALT), and aspartate transaminase (AST) in HUA mice, exerting protective effects on the liver and kidney. Network pharmacology showed that there were 93 common targets for RA, *Poria cocos*, and HUA with the top five core targets tumor necrosis factor (TNF), signal transducer and activator of transcription 3 (STAT3), cysteinyl aspartate specific proteinase 3 (CASP3), jun proto-oncogene (JUN), and estrogen receptor 1 (ESR1). Molecular docking analysis revealed that AG IV, calycosin and formononetin bond well to the core targets.

**Conclusion:**

This research revealed the interaction of RA and FRA with fecal microbiota and Lactobacillus acidophilus, RA and *Poria cocos* were featured with multiple components, target points, and signaling pathways in HUA treatment, which provided fresh insights for further HUA therapeutics.

## 1 Introduction

Hyperuricemia (HUA) is a chronic metabolic disease resulting from decreased excretion, increased uric acid (UA) production, or a combination of both. Resent evidences suggested a pathogenic relationship among HUA, chronic kidney disease, and cardiovascular disease (1). In China, HUA poses a potential public health risk, with adult prevalence at 15.1% (2) and adolescent prevalence, ages 3–19, at 23.3% (3) Traditional Chinese medicine (TCM) confirms a linkage between HUA development and the concept of “dampness evil” (4). The mainstream drugs for the treatment of HUA focus on three types: xanthine oxidase inhibitors, uricosuric drugs, and selective urate reabsorption inhibitor (5), with current clinical options including allopurinol, probenecid, benzbromarone, and lesinurad. However, these medications often have inevitable side effects. Benzbromarone faces restricted usage due to concerns about its potential hepatotoxic effects (6). Probenecid showed limited efficacy and safety in patients which have developed renal impairment (7). To avoid these challenges, some researches turn to TCM to discover a novel treatment for HUA.

Radix Astragali (RA), the dried root of *Astragalus membranaceus* (Fisch.) Bunge, a renowned herbal medicine throughout the world and represents one of the largest flowering plants genuses within the Leguminous family. With over 200 identified compounds, RA is distinguished by its main active compounds notably saponin, flavonoids, and astragalus polysaccharide, which makes RA famous for tonic, hepatoprotective, antimicrobial, and antioxidant characteristics (8). RA was first recorded in Prescriptions for 52 Diseases of the Han Dynasty and has been used in medicine more than 2,000 years (9). According to the Pharmacopeia of the People’s Republic of China (10), it also has functions of replenishing qi, lifting yang, and inducing diuresis to alleviate edema. RA has been verified with the efficacy of improving the kidney function (11) and has confirmed the function in the treatment of HUA (12). *Poria cocos* is the dried sclerotium of *Poria cocos* (Schw.) Wolf and has a long history of medical use in Asian countries (13). It was first recorded in Shennong’s Classic of *Materia Medica*. *Poria cocos* was used to induce diuresis and tonify spleen and be anti-tumor, antioxidant, and anti-inflammatory (10, 14–16). Formulas containing *Poria cocos* such as *Fuling-Zexie* formula and Gegen Juju Fuling formula have been verified with the function of decreasing the level of UA and ameliorating HUA without evident liver and kidney injuries (17, 18). The Huangqi Siwu decoction, which was composed of RA, *Poria cocos*, Angelica sinensis, and other Chinese medicine, has been utilized to treat gout for a long-term (19). Although there have been some advances in RA and *Poria cocos* in the treatment of HUA, the underlying mechanism remains insufficiently elucidated. Previous study showed that bidirectional solid fermentation could augment the efficacy of Chinese medicine, decrease toxicity, and produce new efficacy (20). Author’s research group found that the content of active compounds in FRA was significantly higher than that in RA (21).

The main metabolism of saponin and flavonoids occurs within the enteric tract. The human colonic microbiota is formed from a wide variety of bacteria, which exerts substantial influence on colonic contents metabolism. Some studies have showed that the pivotal role of the colonic microbiota in converting flavonoids and saponin into aglycons, respectively. The colonic microbiota is important in the full accomplishment of the beneficial functions of flavonoids (22–24). In particular, beneficial bacteria play a crucial function in the process, and *Lactobacillus* species are vital and active probiotics within the colon that can provide beneficial metabolites for the host, thereby fostering a stable colonic environment (25). These bacteria are actively engaged in diverse biological processes, including protein biosynthesis, immune regulation, inflammation mediation, gastric mucosa protection, and antioxidation (26, 27). In addition, active compounds in medicine have a positive effect on intestinal flora. Research studies showed that *Bifidobacterium*, *Lactobacillus*, and *Akkermansia* increased in the colon following flavonoid supplementation (28). After rectifying the intestinal microflora imbalance in mice with RA saponin, there was an increase in the abundance of *Bifidobacterium* and *Lactobacillus* (29). HUA may lead to intestinal barrier dysfunction and enhance intestinal permeability (30). Some studies showed that intestinal flora play a significant role in the treatment of HUA. One-third of uric acid was excreted through the gut and was further metabolized by intestinal bacteria (31–33). *Lactobacillus* can express uricase, allantoase, and allantoinase to breakdown UA (34). Bacteroides has powerful anti-hyperuricemia effect in high-protein diet goose (35). Moreover, analyses employing 16S rRNA sequencing validated that the mice in the HUA group displayed an increase in *Lactobacillus* species abundance after administrating with RA (36). While existing studies have confirmed the augmentation of beneficial bacteria abundance after giving RA, knowledge regarding the interaction between RA, FRA, and the colonic microbiota and changes in their metabolism is still insufficient.

Hence, in this study, Radix Astragali was fermented by *Poria cocos* and *in vitro* fecal microbiota colonic fermentation model was constructed to probe the impacts of microbiota on the metabolism of RA and fermented Radix Astragali (FRA). Additionally, fermented RA and FRA with *Lactobacillus acidophilus in vitro* to further delve into the interaction between the compounds and the beneficial bacteria. On the basis of this, a HUA mouse model induced by potassium oxonate (PO) was constructed to figure out the efficacy of FRA and RA in the treatment of HUA. Furthermore, network pharmacology and molecular docking were applied to discuss the underlying mechanisms of RA and *Poria cocos* in the treatment of HUA. This research could provide a scientific foundation for leveraging FRA as a method in the treatment of HUA.

## 2 Materials and methods

### 2.1 Materials

#### 2.1.1 Materials and reagents

RA was obtained from Shanxi Hunyuan Wansheng Huangqi Development Co., Ltd. (Shanxi, China) and validated by Prof. Long Dai of Binzhou Medical University (Yantai, Shandong), and the corresponding voucher specimens (voucher number: 20211001) were stored in the herbarium of Experimental Center of Chinese Medicine of Binzhou Medical University. The RA underwent processing using an 800A disintegrator (Yongkang Hardware and Medical Instrument Plant, China). High-performance liquid chromatography (HPLC)-grade solvents were procured from Tianjin Kemiou Chemical Reagent Co., Ltd. (Tianjin, China). Potato liquid medium and MRS culture medium were sourced from Qingdao Haibo Biotechnology Co., Ltd. (Qingdao, China). Various chemicals such as dipotassium hydrogen phosphate, potassium dihydrogen phosphate, sodium carbonate, potassium persulfate, and sodium hydroxide were acquired from Sinopharm Chemical Reagent Co., Ltd. (Shanghai, China). Salivary amylase, pepsin, gastric lipase, pancreatic enzyme, and pig bile powder were purchased from Shanghai Yuanye Bio-Technology Co., Ltd. (Shanghai, China). Additionally, 1,1-diphenyl-2-picrylhydrazyl (DPPH), 2,2-hydrazine-bis (3-ethylbenzothiazoline-6-sulfonic acid) diamine salt (ABTS), potassium oxonate, and D-fructose were purchased from Shanghai Macklin Biochemical Technology Co., Ltd. (Shanghai, China). Anhydrous ethanol was obtained from Tianjin Yongda Chemical Reagent Co., Ltd. (Tianjin, China). Vanillin was bought from Tianjin Guangfu Fine Chemical Research Institute. Benzbromarone tablets (BT) were procured from Changzhou Kangpu Pharmaceutical Co., Ltd. (Changzhou, China). Biochemical assay kits for UA, triglyceride (TG), alanine aminotransferase (ALT), and aspartate aminotransferase (AST) were obtained from Nanjing Jiancheng Bioengineering Institute, while xanthine oxidase (XOD) assay kits were bought from Beijing Solarbio Science & Technology Co., Ltd. (Beijing, China).

#### 2.1.2 Microbial strains

*Lactobacillus acidophilus* CGMCC1.1878 was acquired from Institute of Microbiology Chinese Academy of Sciences. *Poria cocos* (bio-08656) was acquired from Beijing Bai Ou Bo Wei Biotechnology Co., Ltd. (Beijing, China).

#### 2.1.3 *Poria cocos* solid-state fermentation Radix Astragali

The fermentation of FRA was in accordance with our previous study (21). RA powder and ultrapure water were mixed and sterilized in an YXQ-Sll autoclave (Shanghai Boxun Medical Biological Instrument Co., Ltd., Shanghai, China). Then, the *Poria cocos* seed fermentation broth was inoculated on the former mixture and incubated in a GSP-9160MBE incubator (Shanghai Boxun Medical Biological Instrument) at 27°C for 10 days.

### 2.2 *In vitro* simulation digestion

With slight modifications, *in vitro* simulation digestion procedure was determined in accordance with the published methods in our previous study (21). In the oral phase (OP), 15 g of FRA was combined with 15 mL of phosphoric acid buffer (PBS) and 0.9% normal saline was used to replenish the solution to 180 mL. The pH of the mixture was regulated to 7.0 by adding a 16 mol/L NaOH solution, followed by incubation at 37°C for 10 min in a SHZ-A stable temperature horizontal shaking bath (Changzhou Nuoji Instrument Co., Ltd., Jiangsu, China). Subsequently, 2.588 g of salivary amylase was added, and then, the mixture was digested for 2 min in darkness.

In the gastric phase (GP), the OP sample was replenished with normal saline to 180 mL. The pH was reduced to 2.0 with a 1 mol/L HCl solution, and the mixture was incubated at 37°C for 10 min. Subsequently, 1.491 g of pepsin and 0.671 g of lipase were introduced and the mixture was digested for 2 h in darkness.

In the enteric phase I (EP I), the GP sample was blended with normal saline to 180 mL. The pH increased to 4.7 and the mixture was incubated at 37°C for 10 min. Then, 0.727 g pancreatic enzyme and 0.416 g bile were added. The mixture was digested for 2 h in darkness. In enteric phase II, the EP I sample was mixed with normal saline. The pH of the mixture was increased to 6.5. The sample was mixed with 0.727 g pancreatin and 0.416 g bile. The mixture was digested for 2 h in darkness.

### 2.3 *In vitro* colonic fermentation with fecal microbiota

Four healthy donors (two males and two females) who did not have enteric diseases or accept antibiotics ingestion and adhered to regular eating habits for over 3 months before donation were recruited from Binzhou Medical University. All donors signed the informed consents. This study had been carried out according to the Code of Ethics of the World Medical Association (The Declaration of Helsinki). These experiments were approved by the Medical Ethics Committee of Binzhou Medical University in Shandong, China (ethical approval number: 2024-L029). The procedure of collecting feces did not cause any harms to the donors, and no human experiment was involved. Feces samples were collected and blended evenly; 55 g of mixed feces was added with 550 mL of sterile PBS and was filtered through sterile gauze to obtain 10% w/v fecal slurry. In experimental groups, 10 mL samples of FRA and RA previously experienced *in vitro* simulated digestion were mixed with 15 mL of fecal slurry and 15 mL of GAM in test tubes with stopper, respectively, followed by uniform blending and removal of oxygen using nitrogen. Then, the mixture was incubated in a LAI-3DT anaerobic incubator (Shanghai Longyue Instruments Equipment Co., Ltd., Shanghai, China) in anaerobic conditions at 37°C for a period of time. For the control group, 15 mL of fecal slurry was mixed with 15 mL of GAM and 10 mL of sterile PBS in test tubes with stopper and treated in the same way as experimental groups. At 0 h, 6 h, 12 h, 24 h, and 48 h of fermentation, samples taken from the anaerobic incubator were mixed with 0.1 mL of methanol and centrifuged at 10,000 rpm for 10 min through 3H16RI Intelligent High-Speed Refrigerated Centrifuge (Hunan Herexi Instrument and Equipment Co., Ltd., Hunan, China). The fermentation supernatants at different times were given refrigerated storage. Each group was carried out in triplicate.

### 2.4 *In vitro* colonic fermentation with *Lactobacillus acidophilus*

The fermentation experiment comprised of three groups: FRA, RA, and control groups; 4.5 mL of samples of FRA and RA, which had experienced simulated *in vitro* digestion, were mixed with 4.5 mL of MRS culture medium and 3 mL of *Lactobacillus acidophilus* suspension in test tubes with stopper, respectively. After that, the mixtures were blended evenly. Then, all groups were incubated in the anaerobic incubator at 37 °C. At 0 h, 6 h, 12 h, 24 h, and 48 h of fermentation, samples were mixed with 0.1 mL of methanol and centrifuged at 10,000 rpm for 10 min. The fermentation supernatants from different times were refrigerated. Each group was carried out in triplicate.

### 2.5 Enumeration of bacteria

The viable cell counts were quantified by plate count. One milliliter of samples of *in vitro* colonic fermentation of *Lactobacillus acidophilus* at 0 h, 12 h, 24 h, 36 h, and 48 h was diluted by PBS until 10^6^ dilutions, respectively. Fifty microliters of the dilutions was evenly smeared on MRS agar. All plates were incubated at 37 °C under an anaerobic environment for 48 h.

### 2.6 Antioxidant capacity assay

The *in vitro* antioxidant capacity of the samples was determined according to the method of our previous study (21). In this experiment, we evaluated the antioxidant capacity of vitamin C (Vc) and the product of the *in vitro* fecal microbiota colonic fermentation at 0 h and 48 h through DPPH and ABTS radical scavenging assays.

#### 2.6.1 Sample preparation

Supernatants attained from *in vitro* colonic fermentation with fecal microbiota at 0 h and 48 h were distilled to constant weights in a vacuum environment using a RE-52AA rotary evaporator device (Shanghai Yarong Biochemical Instrument Factory, Shanghai, China). The solids then were weighted.

#### 2.6.2 DPPH radical scavenging assay

The antioxidant activities of the samples were carried out through the DPPH free radical scavenging assay. One milligram per milliliter sample solution of 1,600 μL, 800 μL, 400 μL, 200 μL, 100 μL, and 100 μL diluted solutions was mixed with 1 mL DPPH, respectively, and then blended with anhydrous ethanol to 4 mL. After a 30-min incubation in darkness, the absorbance of the mixed solutions was measured at 517 nm utilizing TU-1810 APC UV–Visible spectrophotometer at Beijing Persee General Instrument Co., Ltd. (Beijing, China). The free radical scavenging activities were evaluated according to the following formula:


$$\text{DPPH}\%=\left(A_{3}+A_{2}-A_{1}\right)∕A_{3}\times 100\%$$

where *A*_1_ represents the absorbance of the reaction mixture, *A*_2_ represents the absorbance of the blank (without DPPH), and *A*_3_ represents the absorbance of the control (without sample).

#### 2.6.3 ABTS radical scavenging assay

The antioxidant activities of the samples were measured through ABTS radical cation (ABTS^+^) scavenging activity. Different concentrations of sample solutions 0.1 mL were mixed with 4 mL ABTS^+^ working solution and incubated in darkness for 6 min. The mixture absorbance was measured at 734 nm utilizing TU-1810 APC UV–Visible spectrophotometer. The free radical scavenging activities were assessed according to the following equation:


$$\text{ABTS}\%=\left[A_{0}-\left(A-B\right)\right]∕A_{0}\times 100\%$$

where *A*_0_ represents the absorbance of the control (without sample), *A* represents the absorbance of the reaction mixture, and *B* represents the absorbance of different concentrations of the sample solution.

### 2.7 Pharmacodynamic experiment of anti-hyperuricemia

In this study, an HUA mouse model was constructed by the co-treatment with potassium oxonate and water infused with fructose. After consecutive 24-day experiments, biochemical assays were detected and the results were analyzed.

#### 2.7.1 Animal models

All animal experiments were performed as approved by the ARRIVE guidelines and the Guidelines for Care and Use of Laboratory Animals, and the animal experiments were approved by the Animal Ethics Committee of Binzhou Medical University in Shandong, China (ethics approval number: 2024-L030) and complied with the principles of 3R. Male Kunming mice (weight range: 25–30 g) were procured from Jinan Pengyue Laboratory Animal Breeding Limited Company (laboratory animal license number: SCXK (Lu) 20190003). The mice were reared in a specific pathogen-free laboratory characterized by a room temperature of 24 ± 2°C, humidity of 60 ± 5%, and a 12 h light/dark cycle. All mice were acclimated to this environment for 3 days prior to the experiment procedure. The mice were randomly divided into six groups (*n* = 8), namely, the normal control group (NC), the HUA group (PO), the positive group (PO + BT), the RA group (PO + RA), the FRA group (PO + FRA), and the *Poria cocos* group (PO + Pc).

Except for the control, all groups were intragastrically injected with 500 mg/kg potassium oxonate once a day to construct the HUA model, which was carried out in accordance with the previous study (37). Mice in the positive group, RA group, FRA group, and *Poria cocos* group were given benzbromarone (10 mg/kg), RA (2.5 g/kg), FRA (2.5 g/kg), *Poria cocos* (2.5 g/kg) 1 h after modeling for 24 consecutive days, respectively. The dose of benzbromarone was referred to the previous study (38). The doses of RA, FRA, and *Poria cocos* were calculated according to the Methodology of Pharmacological Experiment (39). The NC group received distilled water, and the rest received 10% fructose-drinking water. All mice drank and ate freely according to the guidelines set by the China Animal Protection Association.

Mice from six groups were observed and the index of the mice was recorded, which containing the condition of eating, drinking, mental state, and the change in their weights. Orbital blood samples were collected 3 h after administration on 12 d and 24 d and were centrifuged at 3,500 rpm for 10 min and then stored at-20°C refrigerator. The mice fasted after administration on day 24 were anesthetized 3 h after the last administration and then were euthanized by cervical dislocation. The kidneys and livers of the mice were removed and immersed in 4% paraformaldehyde for subsequent use.

#### 2.7.2 Biochemical assays

Assay kits and ELISA were utilized to measure serum levels of UA, TG, XOD, ALT, and AST.

#### 2.7.3 Histological analysis

The tissues of kidneys and livers were fixed in 4% paraformaldehyde, then cut into histopathological slices and stained with hematoxylin and eosin (H & E), and then examine the slices with an optical microscope at 200× magnification.

### 2.8 Content determination

The content determination of the samples was carried out according to the methods of our previous study (21).

#### 2.8.1 Determination of total saponins content

Different volumes of AG IV standard solutions (0.5 mg/mL) were added to test tubes with stoppers and were replenished with methyl alcohol to 0.5 mL, mixing with 0.5 mL of 8% vanillin ethanol solution and 5 mL of 72% sulfuric acid. After incubating at 62°C for 20 min and cooling to room temperature, the absorbance of solutions was measured at 540 nm using TU-1810 APC UV–Visible spectrophotometer to establish a standard curve. Sample solutions (0.1 mL) prepared in sections 2.3 and 2.4 were determined as the method described above.

#### 2.8.2 Determination of total flavonoids

The different volumes of the Rutin standard solution (0.2 mg/mL) were added into 10 mL volumetric flasks. Moreover, the Rutin solutions were mixed with 70% ethanol, 5% NaNO_2_ solution, 10% Al(NO_3_)_3_ solution, and 4% NaOH solution, then diluting to volume with 70% ethanol. The absorbance of mixture was measured at 510 nm using TU-1810 APC UV–Visible spectrophotometer. Then, the standard curve was drawn. The sample solutions (1.5 mL) prepared in sections 2.3 and 2.4 were added to 10-mL volumetric flasks and repeated the same method described above.

#### 2.8.3 HPLC method

An ELSD-16 (SHIMADZU, Japan) and a DGU-20A3R HPLC system (SHIMADZU, Japan) with a Phenomenex C18 column (4.60 × 250 mm, 5 μm) were utilized to determine AG IV content. The mobile phases were comprised of acetonitrile (A) and water (B) with a gradient elution as follows: 0-20 min, 96-80% B; 20-40 min, 80-70% B; 40-65 min, 70-40% B; 65-70 min, 40-96% B; 70-80 min, and 96% B, and the flow rate was at 1.0 mL/min with 20 μL of injection volume under 25°C.

The content of calycosin was determined by HPLC, with a Thermo Scientific Dionex Ultimate 3000 series HPLC system (Dionex, USA), a diode array detector (Dionex, USA), and a Kromasil 100-5-C18 column (4.6 by 250 mm). The mobile phases were comprised of 0.05% formic acid (A) and acetonitrile (B) with a gradient elution as follows: 0-5 min, 0-5% B; 5-10 min, 5-20% B; 10-25 min, 20-25% B; 25-35 min, 25-30% B; 35-40 min, 30-35% B; 40-54 min, 35-40% B; 54-55 min, 40-0% B, and the flow rate was at 1.0 mL/min with 10 μL of injection volume under 30°C.

### 2.9 Network pharmacology analysis

#### 2.9.1 Screening of active compounds and effect target of RA and *Poria cocos*

Retrieving compound information of RA and *Poria cocos* from the TCMSP database,^1^ focusing specifically on oral bioavailability (OB) ≥ 30% and drug-likeness (DL) ≥ 0.18 (40). Subsequently, low-intestinal-activity compounds were eliminated through the SwissADME website. The English names of the active compounds were imported into the PubChem database. Afterward, retrieving SMILES and the Swiss Target Prediction platform^2^ was employed to predict targets with a possibility ≥0 and then entailed screening and collecting targeted compounds targets.

#### 2.9.2 Screening of HUA disease targets

“Hyperuricemia” was utilized as a key word to collect the HUA disease targets from the following databases, such as GeneCards,^3^ DisGeNET,^4^ and MOIM.^5^ And the targets attained from these databases were merged and the repeating items were eradicated.

#### 2.9.3 Screening of drug-disease common targets

The targets of RA, *Poria cocos*, and HUA were imported into Draw Venn Diagram tool, respectively, extracting common targets among them and painted the Venn diagram.

#### 2.9.4 Construction of drug component target interaction network

The information of the components and the data of the targets was imported into Cytoscape 3.9.1 to construct an interaction network diagram of “drug component target.”

#### 2.9.5 Construction of protein–protein interaction network

A protein–protein interaction (PPI) network was devised utilizing the STRING^6^ online platform database. The function of Centiscape 2.2 in Cytoscape 3.9.1 was used to extract the core targets by calculating degree values, betweenness centrality and closeness centrality.

#### 2.9.6 GO and KEGG enrichment pathway analysis

Gene Ontology (GO) enrichment pathway analysis was executed through the DAVID database to explore molecular function (MF), biological process (BP), cellular component (CC), and other gene target functions. Kyoto Encyclopedia of Genes and Genomes (KEGG) enrichment pathway analysis was carried out to discuss the biological pathways of the drug function and analyze the pathway enrichment of the potential targets. The bioinformatics platform was used to show visual analysis.

#### 2.9.7 Molecular docking

The 3D structure of the core target protein was sourced from the Protein Database,^7^ serving as protein receptor, and PyMOL was used to remove the ligand and non-protein from the target protein and saved the consequence as a pdb format file. Hydrogen atoms were added to protein receptors with AutoDock Tools 1.5.7, and then, the structure was saved as a pdbqt format file. The 2D structure of AG IV, calycosin, and formononetin was retrieved from PubChem database^8^ and saved as a mol2 format file. We applied AutoDockTools1.5.7 to add hydrogen atoms and examine central node of the molecules and the rotatable keys, saving as a pdbqt format file and served as ligands. AutoDock Vina 1.1.2 was utilized to carry out molecular docking and assessed the binding energy between the ligands and protein receptors, determining the binding activity. PyMOL software was used to perform visual display.

### 2.10 Statistical analysis

The collection of experimental data was done using Excel 2019 and SPSS software version 26.0 was used to carry out statistics analysis. Quantitative data are presented as the mean ± SD of three independent experiments. Significant differences among the data were analyzed by ANOVA. Statistical significance was established if the *p*-value was less than 0.05.

## 3 Results and discussion

### 3.1 The results of *in vitro* colonic fermentation with fecal microbiota

RA and FRA after *in vitro* digestion passed through *in vitro* fecal microbiota colonic fermentation in an anaerobic environment at 37°C with sampling at 0 h, 6 h, 12 h, 24 h, and 48 h of the fermentation. Table 1 presents the alternations in the contents of the compounds during the *in vitro* colonic fermentation. The contents of the main compounds in two groups experienced a decline throughout the fermentation process. The AG IV content in FRA was 1.46 times than that found in RA at the initial stage, and in FRA, it was higher than in RA throughout the fermentation. At 48 h, the AG IV contents in FRA and RA had decreased to 0.60 ± 0.10 mg/g and 0.47 ± 0.04 mg/g, respectively, representing decrease of 52.76 and 45.98% from the initial value (*p* < 0.05). The calycosin contents remained relatively stable over fermentation time. Compared to the initial stage, at 48 h, the total saponins contents in FRA and RA reduced to 54.00 ± 2.11 mg/g and 43.31 ± 3.55 mg/g, representing decrease of 45.60 and 58.26%, respectively (*p* < 0.05). At 48 h of fermentation, the contents of total flavonoids decreased from 13.63 ± 0.41 mg/g to 11.54 ± 1.33 mg/g in FRA and from 10.13 ± 0.50 mg/g to 7.04 ± 0.05 mg/g in RA, respectively. The main metabolism pathways of microbiota include hydrolysis, ring cleavage and dihydroxylation (41). The reduction in AG IV may be attributed to deglycosylation, or its conversion into cycloastragalool-6-*β*-D-glucopyranoside, saponins cycloastragalool, or other smaller molecular forms (42). The reduction in total saponins is likely due to deglycosylation, and the compound was converted into secondary metabolites and ultimately into aglycone (43).

**Table 1:** Content of compounds of FRA and RA *in vitro* colonic fermentation with fecal microbiota.

	Compounds (mg/g)	0 h	6 h	12 h	24 h	48 h
FRA	AG IV	1.27 ± 0.09^a^	0.89 ± 0.08^b^	0.76 ± 0.16^bc^	0.69 ± 0.03^bc^	0.60 ± 0.10^c^
	Calycosin	0.18 ± 0.03^ab^	0.19 ± 0.01^a^	0.15 ± 0.00^ab^	0.14 ± 0.01^b^	0.16 ± 0.00^ab^
	Total saponins	99.26 ± 2.25^a^	78.97 ± 6.30^b^	85.57 ± 10.48^b^	66.39 ± 4.28^bc^	54.00 ± 2.11^d^
	Total flavonoids	13.63 ± 0.41^a^	12.57 ± 0.21^ab^	11.45 ± 0.65^bc^	10.45 ± 0.86^c^	11.54 ± 1.33^bc^
RA	AG IV	0.87 ± 0.06^a^	0.86 ± 0.02^a^	0.55 ± 0.01^b^	0.45 ± 0.03^c^	0.47 ± 0.04^bc^
	Calycosin	0.17 ± 0.01^bc^	0.23 ± 0.01^a^	0.16 ± 0.00^c^	0.17 ± 0.01^bc^	0.18 ± 0.00^b^
	Total saponins	103.76 ± 5.05^a^	82.14 ± 3.19^b^	61.38 ± 0.31^d^	74.37 ± 4.70^c^	43.31 ± 3.55^e^
	Total flavonoids	10.13 ± 0.50^a^	8.65 ± 1.84^bc^	8.91 ± 0.23^bc^	8.33 ± 0.51^bc^	7.04 ± 0.05^c^

FRA, fermented Radix Astragali; RA, Radix Astragali; AG IV, Astragaloside IV.

Values are expressed as mean ± standard error from three replications. Means with different superscripts in the same row show significant difference (*p* < 0.05).

**Figure 1:**
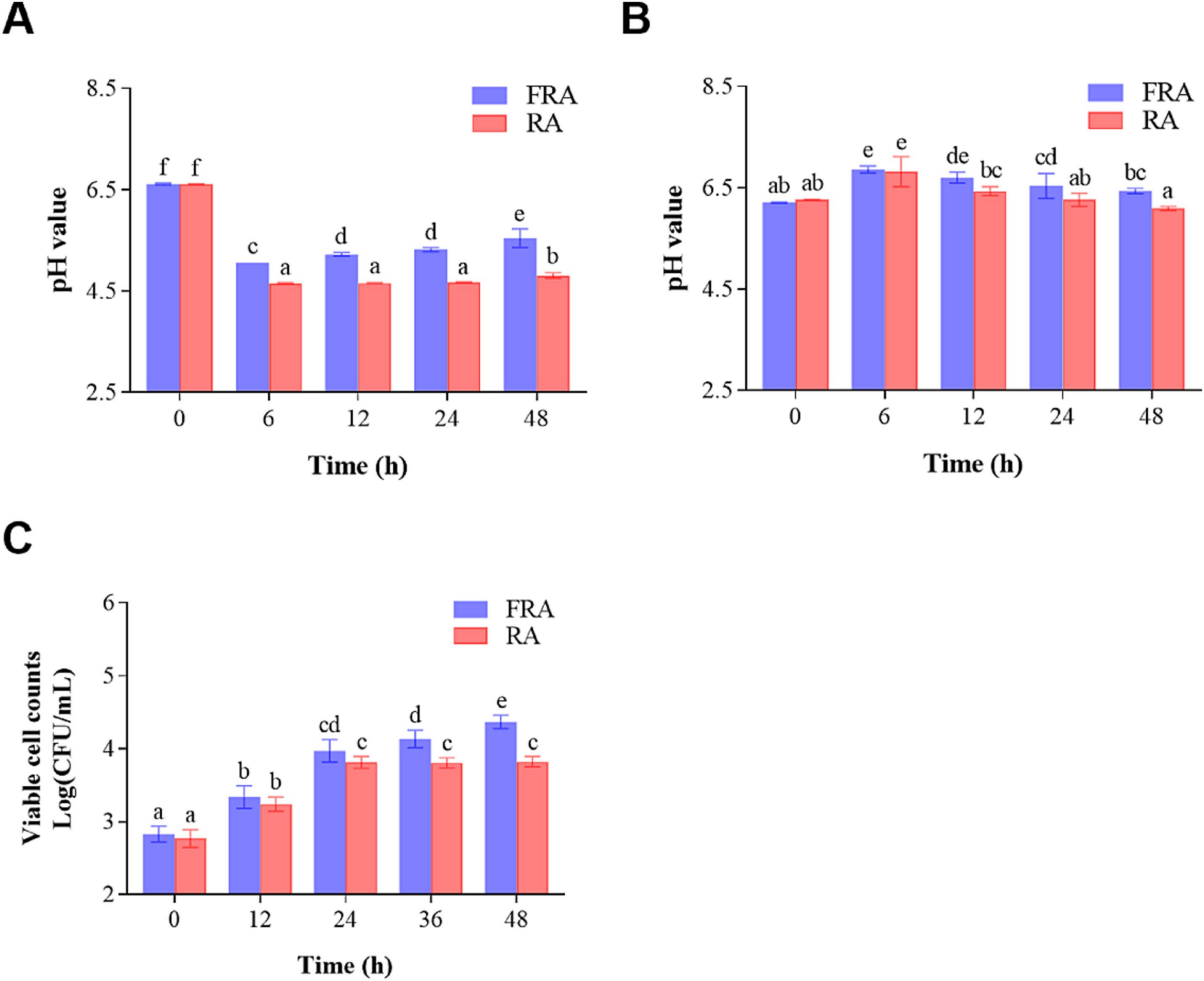
**(A)** Change in pH during *in vitro* colonic fermentation of the fecal microbiota. **(B)** Change in pH during *in vitro* colonic fermentation of *Lactobacillus acidophilus*. **(C)** Viable cell counts of *Lactobacillus acidophilus* before and after fermentation.

Figure 1A illustrates the alternations in pH values during *in vitro* colonic fermentation. The pH values of the FRA and the RA group were 6.61 ± 0.03 and 6.61 ± 0.01 at 0 h, respectively. Over the course of 6 h, the pH values in FRA and RA decreased to 5.06 ± 0.00 and 4.66 ± 0.02, performing a reduction of 23.45 and 29.50% compared to the initial stage, respectively (*p* < 0.05). The reduction in pH may be attributed to the production of polysaccharides and oligosaccharides, or the generation of short-chain fatty acids (SCFAs) by the fecal microbiota (44). A study indicated that the increase in SCFA production could lower pH values during fermentation process (45). After 6 h, the pH values of the FRA group experienced a slight and consecutive increase. That’s may be due to the selective effect of FRA on beneficial microbiota in feces to allow them to facilitate the pH adjustments within the colonic environment (46). However, the pH values in RA remained stable after 6 h fermentation.

### 3.2 The results of *in vitro* colonic fermentation with *Lactobacillus acidophilus*

Previous studies have shown that the abundance of *Lactobacillus* increased after giving RA to HUA mice (36). In this study, *Lactobacillus acidophilus* was chosen to ferment FRA and RA exploring the interactive effects between compounds and probiotics. RA and FRA after *in vitro* digestion were collected at 0 h, 6 h, 12 h, 24 h, and 48 h and passed through *in vitro* colonic fermentation with *Lactobacillus acidophilus* under anaerobic conditions at 37°C.

Table 2 presents the changes observed during the fermentation. Initial observations revealed a higher content of AG IV in FRA compared to RA, possibly due to the metabolism or biotransformation of other astragalosides acting as precursors in the fermentation caused by *Poria cocos* and the *in vitro* digestion process. The contents of AG IV in FRA and RA increased during the first 12 h and decreased during the following time, peaking at 12 h with values of 1.14 ± 0.20 mg/g and 0.63 ± 0.05 mg/g (*p* < 0.05), respectively, may be due to the biotransformation of other astragalosides as precursors under the role of *Lactobacillus acidophilus* (47). The following decrease may be due to enzymes activity by *Lactobacillus acidophilus*, leading to the conversion of AG IV into secondary metabolites or other small molecules. The calycosin contents remained relatively stable over fermentation time, whereas total saponins contents exhibited an initial rise followed by a decrease in both groups. The content of total saponins reached a peak of 136.34 ± 6.15 mg/g at 12 h in FRA, which was an increase of 282.01% compared to the initial (*p* < 0.05), and in RA, a peak of 115.12 ± 4.12 mg/g at 24 h, presenting a 221.74% increase from the initial (*p* < 0.05). In FRA, a reduction in total flavonoids was observed after the peak value 6.35 ± 0.06 mg/g at 6 h. In RA, the total flavonoids content decreased from 4.28 ± 0.03 mg/g to 1.91 ± 0.18 mg/g at 48 h. The reduction in total flavonoids may due to deglycosylation of hydrolytic enzymes produced by *Lactobacillus acidophilus* (48). Under the influence of *Lactobacillus acidophilus*, the contents of AG IV, total saponins, and total flavonoids rose first and then declined in FRA group, and in RA group, the contents of total saponins and AG IV increased at the beginning and then fell.

**Table 2:** Content of compounds of FRA and RA *in vitro* colonic fermentation with *Lactobacillus acidophilus*.

	Compounds (mg/g)	0 h	6 h	12 h	24 h	48 h
FRA	AG IV	0.59 ± 0.05^bc^	0.89 ± 0.05^ab^	1.14 ± 0.20^a^	0.76 ± 0.01^bc^	0.50 ± 0.19^c^
	Calycosin	0.16 ± 0.01^a^	0.14 ± 0.01^b^	0.15 ± 0.00^ab^	0.14 ± 0.00^b^	0.15 ± 0.01^ab^
	Total saponins	35.69 ± 1.27^d^	78.57 ± 2.03^c^	136.34 ± 6.15^a^	87.24 ± 2.05^b^	25.83 ± 1.26^c^
	Total flavonoids	5.58 ± 0.57^b^	6.35 ± 0.06^a^	6.11 ± 0.06^ab^	2.62 ± 0.07^c^	3.03 ± 0.09^c^
RA	AG IV	0.35 ± 0.00^c^	0.47 ± 0.04^b^	0.63 ± 0.05^a^	0.31 ± 0.02^c^	0.32 ± 0.01^c^
	Calycosin	0.10 ± 0.00^c^	0.15 ± 0.01^b^	0.15 ± 0.02^b^	0.16 ± 0.01^b^	0.21 ± 0.01^a^
	Total saponins	35.78 ± 0.28^d^	68.49 ± 1.21^c^	82.76 ± 2.37^b^	115.12 ± 4.12^a^	31.56 ± 0.98^e^
	Total flavonoids	4.28 ± 0.03^a^	2.97 ± 0.14^b^	2.69 ± 0.73^bc^	2.12 ± 0.09^cd^	1.91 ± 0.18^d^

FRA, fermented Radix Astragali; RA, Radix Astragali; AG IV, astragaloside IV.

Values are expressed as mean ± standard error from three replications. Means with different superscripts in the same row show a significant difference (*p* < 0.05).

Figure 1B illustrates the changes in the pH values of the *in vitro* colonic fermentation of *Lactobacillus acidophilus*. The pH values of two groups increased within the initial 6 h and then went down. At 0 h of fermentation, the pH values of the FRA and the RA groups were 6.21 ± 0.02 and 6.27 ± 0.01, respectively. Subsequently, at 6 h of the fermentation, the pH values of the FRA and the RA peaked at 6.86 ± 0.10 and 6.82 ± 0.42, performing increases of 10.47 and 8.77% compared to the initial (*p* < 0.05), respectively. The reduction in pH values in both groups after 6 h of fermentation suggests a plausible connection to the production of SCFAs by *Lactobacillus acidophilus* as the fermentation processes (49).

To further probe the influence of RA and FRA on *Lactobacillus acidophilus*, viable cell counts were monitored throughout the fermentation process, which was shown in Figure 1C. During the fermentation process, FRA continuously exhibited higher viable cell counts compare to RA. As the colonic fermentation time went by, the viable cell counts in FRA held fiercely increasing trend before 24 h and increased slowly after 24 h. In the FRA, the initial viable cell count was 2.83 ± 0.11 Log (CFU/mL) and augmented to 4.37 ± 0.09 Log (CFU/mL) through 48-h fermentation process, revealing an increase of 54.42% compared to the initial (*p* < 0.05). The initial viable cell counts in RA were 2.77 ± 0.12 Log (CFU/mL) at 0 h, increasing to 3.81 ± 0.08 Log (CFU/mL) at 24 h of fermentation (*p* < 0.05) and stayed a steady trend after 24 h. A study demonstrated that *Bacillus subtilis*-fermented *Astragalus membranaceus* could increase the abundances of butyrate-producing bacteria and probiotics (50), which is accordance with the results of the study. Another study showed that *Lactobacillus plantarum* fermented *Astragalus* could reshape the richness and diversity of microbiota in and increased the abundance of *Lactobacillus* in gut (51). The result of this study showed that both FRA and RA could increase viable cell counts of *Lactobacillus acidophilus* and FRA was more conducive to the growth of probiotic bacteria compared to RA.

### 3.3 DPPH and ABTS of the product of fermentation of fecal microbiota

The products antioxidant activities of *in vitro* fecal microbiota colonic fermentation were assessed by DPPH and ABTS radical scavenging rates, with Vc serving as a control reference. The antioxidant activities of FRA and RA were related to the concentration of the samples, and the antioxidant activity of Vc rose fiercely with increasing samples concentration.

**Figure 2:**
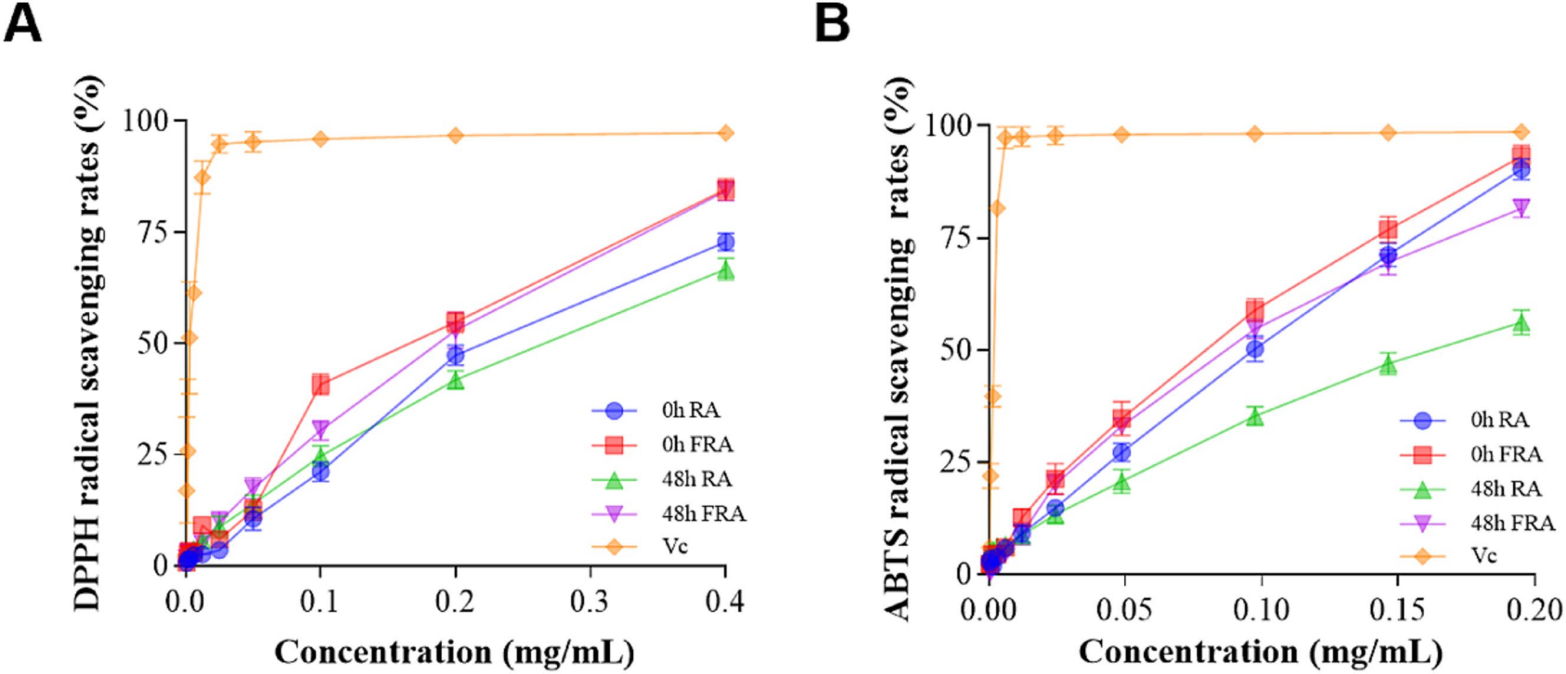
**(A)** DPPH radical scavenging rate. **(B)** ABTS radical scavenging rate.

The DPPH antioxidant results are shown in Figure 2A. The DPPH scavenging activity of 0 h FRA was higher than 0 h RA, 48 h FRA, and 48 h RA. In particular, at the concentration of 0.4 mg/mL, the descending order of DPPH free radical scavenging rates was as follows: Vc 97.33%, 0 h FRA 84.68%, 48 h FRA 84.25%, 0 h RA 72.82%, and 48 h RA 66.75%.

The ABTS antioxidant results are shown in Figure 2B. The ABTS scavenging activity of 0 h FRA was strikingly higher than 48 h FRA, 0 h RA, and 48 h RA. At the concentration of 0.2 mg/mL, the descending order of ABTS free radical scavenging rates was as follows: Vc 98.60%, 0 h FRA 93.12%, 0 h RA 90.29%, 48 h FRA 81.52%, and 48 h RA 56.14%.

Both the DPPH and ABTS results preformed that the antioxidant capacity of the FRA is superior to that of RA. Additionally, the figures illustrate that the antioxidant capacity of the samples increased with increasing concentration.

### 3.4 Effect of RA, FRA, and *Poria cocos* on hyperuricemia in mice

In this experiment, six groups of Kunming mice were utilized to successfully constructed an HUA model. Moreover, the final results show that RA, FRA, and *Poria cocos* had different pharmacodynamic effects on the treatment of HUA.

#### 3.4.1 Effect of different treatment on UA, TG, XOD, ALT, and AST levels in HUA mice

**Figure 3:**
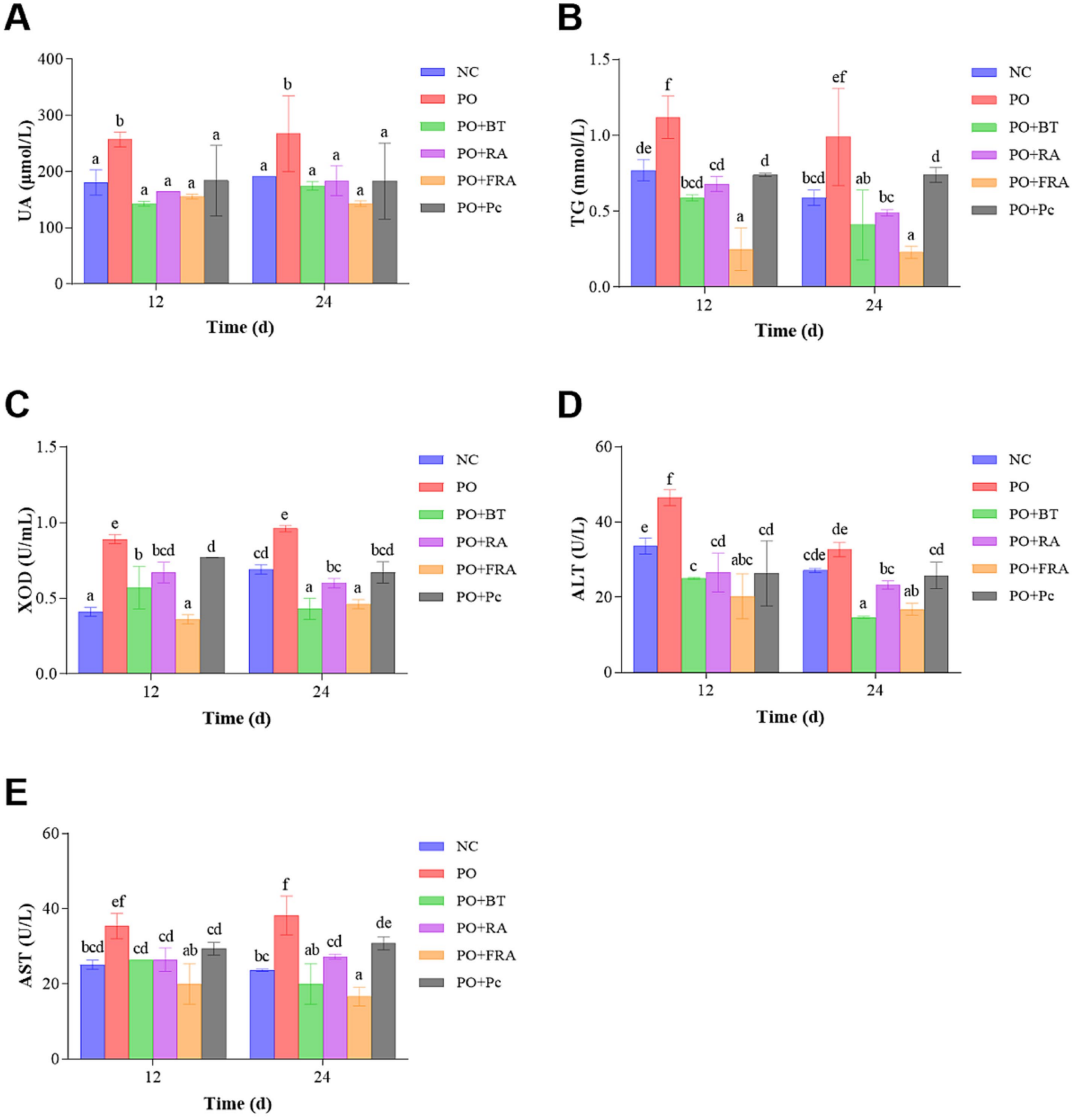
Serum levels of biochemical ingredients: **(A)** UA, **(B)** TG, **(C)** XOD, **(D)** ALT, and **(E)** AST.

The level of UA served as a key indicator of hyperuricemia and was a significant judge of the success of the model. As shown in Figure 3A, at 24 d, the UA level in the HUA group was 267.61 ± 67.72 μmol/L and 1.40 times as much as the 191.55 ± 0.00 μmol/L observed in the NC group (*p* < 0.05), indicating the successful construction of the HUA model. After 24 d of treatment, the UA levels in the positive, RA, FRA, and *Poria cocos* groups were significantly reduced to 174.45 ± 7.69 μmol/L, 183.95 ± 26.68 μmol/L, 143.08 ± 4.81 μmol/L, and 183.09 ± 67.72 μmol/L, representing decreases of 34.81, 31.26, 46.53, and 31.58% compared to the HUA, respectively (*p* < 0.05). The results show that FRA demonstrates the most striking effect on promoting UA excretion.

As can be observed in Figure 3B, the TG levels in the HUA group were 0.99 ± 0.32 mmol/L and 1.68 times as much as the NC group, which was 0.59 ± 0.05 mmol/L at 24 d (*p* < 0.05). After 24 d treatment, the TG levels in the positive, RA, FRA, and *Poria cocos* groups experienced striking decreases to 0.41 ± 0.23 mmol/L, 0.49 ± 0.02 mmol/L, 0.23 ± 0.04 mmol/L, and 0.74 ± 0.05 mmol/L, representing decreases of 58.59, 50.51, 76.77, and 25.25% compared to the HUA, respectively (*p* < 0.05). TG level in FRA exhibits the most substantial decrease.

As can be observed in Figure 3C, after 24 d of consecutive treatment, the XOD level in the HUA group was 0.96 ± 0.02 U/mL, 1.39 times as much as the NC group’s 0.69 ± 0.03 U/mL (*p* < 0.05). After 24 d treatment, the XOD levels in the positive, RA, FRA, and *Poria cocos* groups were 0.43 ± 0.07 U/mL, 0.60 ± 0.03 U/mL, 0.46 ± 0.03 U/mL, and 0.67 ± 0.07 U/mL, reflecting decreases of 55.21, 37.50, 52.08, and 30.21% compared to the HUA, respectively (*p* < 0.05). The result suggests that the efficacy of FRA in reducing UA was related to restricting the XOD levels and inhibiting UA production. Benzbromarone performed its functions mainly by decreasing reabsorption of UA through inhibiting the URAT1 in kidney tubules (52), while our result indicated that benzbromarone could impact XOD activity, which is in accordance with a previous study (53).

As can be observed in Figure 3D, the ALT level in the HUA group demonstrated a 1.20-fold increase at 32.68 ± 1.89 U/L compared to the NC group’s 27.14 ± 0.54 U/L at 24 d (*p* < 0.05). After 24 d of treatment, the ALT levels in the positive, RA, FRA, and *Poria cocos* groups decreased to 14.68 ± 0.27 U/L, 23.29 ± 1.09 U/L, 16.78 ± 1.62 U/L, and 25.79 ± 3.53 U/L, representing decreases of 55.08, 28.73, 48.65, and 21.08% compared to the HUA, respectively (*p* < 0.05).

As can be seen in Figure 3E, the AST level in the HUA group showed a 1.62-fold increase at 38.21 ± 5.17 U/L compared to the NC group’s 23.62 ± 0.29 U/L at 24 d (*p* < 0.05). After 24 d consecutive treatment, the AST levels in the positive, RA, FRA, and *Poria cocos* groups were 20.02 ± 5.37 U/L, 27.27 ± 0.63 U/L, 16.61 ± 2.49 U/L, and 30.78 ± 1.71 U/L, indicating decreases of 47.61, 28.63, 56.53, and 19.45% compared to the HUA, respectively (*p* < 0.05). These results suggest that FRA was likely to reverse liver damage resulting from HUA.

#### 3.4.2 Effect of RA, FRA, and *Poria cocos* on kidney and liver pathological changes

**Figure 4:**
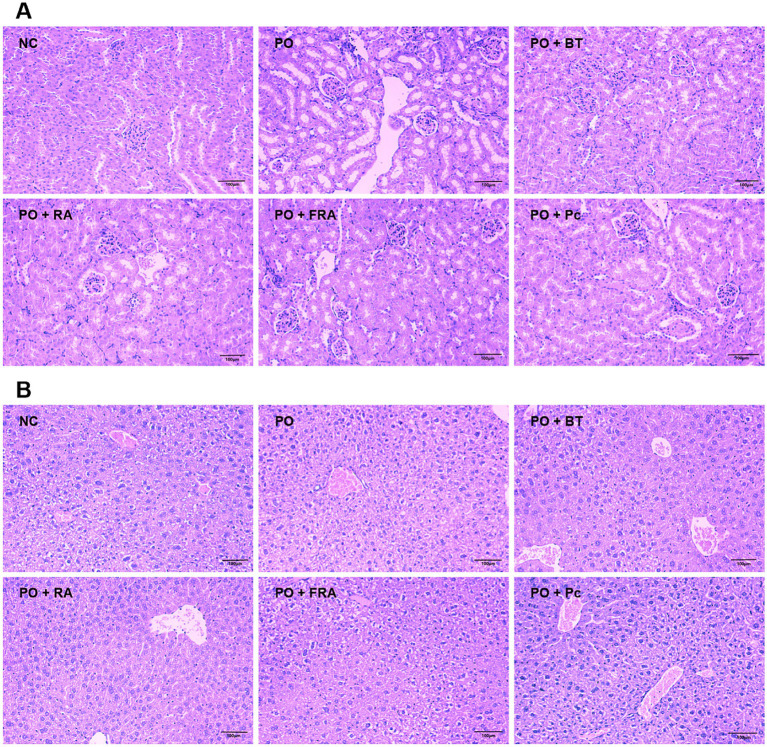
Pathological changes for PO-induced HUA mice. **(A)** Kidney histopathology (magnification: ×200, scale bar: 100 μm). **(B)** Liver histopathology (magnification: ×200, scale bar: 100 μm). NC, normal control; PO, potassium oxonate; BT, benzbromarone tablet; RA, Radix Astragali; FRA, *Poria cocos* solid fermenting Radix Astragali; Pc, *Poria cocos* (*n* = 8 mice per group).

In Figure 4A, the kidney tissues from the control display a healthy structure with regularly arranged glomeruli, normal renal tubular epithelial cells, and intact medulla kidney. While the HUA and the *Poria cocos* group exhibit several abnormal changes, including obvious renal tubule dilation, hydropic degeneration of renal tubular epithelial cells, and inflammatory cell infiltration around the local blood vessel. The positive RA and FRA groups attenuated kidney damage and ameliorated hydropic degeneration of renal tubular epithelial cells. These findings demonstrate that RA and FRA could improve kidney histopathological alternations in mice, thus alleviating the adverse effects of HUA.

As shown in Figure 4B, the liver tissues hepatocytes in the control exhibit a normal condition. While the HUA and the *Poria cocos* group perform severe watery and vacuolar degeneration within the hepatocytes. Treatment with benzbromarone, RA, and FRA show minor watery degeneration of the hepatocytes without inflammatory infiltration. These results indicate that RA and FRA hold the ability to ameliorate the histological changes in hepatocytes induced by HUA, offering beneficial effects on liver tissues.

Collectively, FRA displayed the most striking pharmacodynamic effects on the reduction in UA, TG, and AST levels. Compared with RA and *Poria cocos*, FRA could remarkably reduce the serum level of XOD and ALT. Meanwhile, RA and FRA could improve the kidney and liver pathological changes caused by HUA. The results are in accordance with other studies on *Astragalus membranaceus* protection in the kidney (50, 53, 54).

### 3.5 Network pharmacology analysis

#### 3.5.1 Main active compounds and effect target of RA–*Poria cocos*

Table 3 demonstrates the selection of 18 active compounds in RA and 8 in *Poria cocos* based on DL and OB. A total of 544 active compounds and effect targets were obtained through the Swiss Target Prediction website.

**Table 3:** Main active ingredients of RA and *Poria cocos* obtained from TCMSP database.

Traditional Chinese Medicine	Mol ID	Compounds	Oral bioavailability (OB%)	Drug likeness (DL)
RA	MOL000391	Ononin	11.52	0.78
	MOL000239	Jaranol	50.83	0.29
	MOL000401	Astragaloside I	46.79	0.11
	MOL000403	Astragaloside II	46.06	0.13
	MOL000354	Isorhamnetin	49.60	0.31
	MOL000371	3,9-di-O-methylnissolin	53.74	0.48
	MOL000405	Astragaloside III	31.83	0.10
	MOL000378	7-O-methylisomucronulatol	74.69	0.30
	MOL000379	9,10-dimethoxypterocarpan-3-O-β-D-glucoside	36.74	0.92
	MOL000380	(6aR,11aR)-9,10-dimethoxy-6a,11a-dihydro-6H-benzofurano [3,2-c] chromen-3-ol	64.26	0.42
	MOL000387	Bifendate	31.10	0.67
	MOL000392	Formononetin	69.67	0.21
	MOL000398	Isoflavanone	109.99	0.30
	MOL000417	Calycosin	47.75	0.24
	MOL000422	Kaempferol	41.88	0.24
	MOL000438	(3R)-3-(2-hydroxy-3,4-dimethoxyphenyl) chroman-7-ol	67.67	0.26
	MOL000407	Astragaloside IV	22.50	0.15
	MOL000098	Quercetin	46.43	0.28
*Poria cocos*	MOL000273	(2R)-2-[(3S,5R,10S,13R,14R,16R,17R)-3,16-dihydroxy-4,4,10,13,14-pentamethyl-2,3,5,6,12,15,16,17-octahydro-1H-cyclopenta [a] phenanthren-17-yl]-6-methylhept-5-enoic acid	30.93	0.81
	MOL000279	Cerevisterol	37.96	0.77
	MOL000283	Ergosterol peroxide	40.36	0.81
	MOL000287	3beta-Hydroxy-24-methylene-8-lanostene-21-oic acid	38.70	0.81
	MOL000289	Pachymic acid	33.63	0.81
	MOL000292	Poricoic acid C	38.15	0.75
	MOL000296	Hederagenin	36.91	0.75
	MOL000300	Dehydroeburicoic acid	44.17	0.83

#### 3.5.2 Retrieving of disease targets and screening of active components-disease common targets

**Figure 5:**
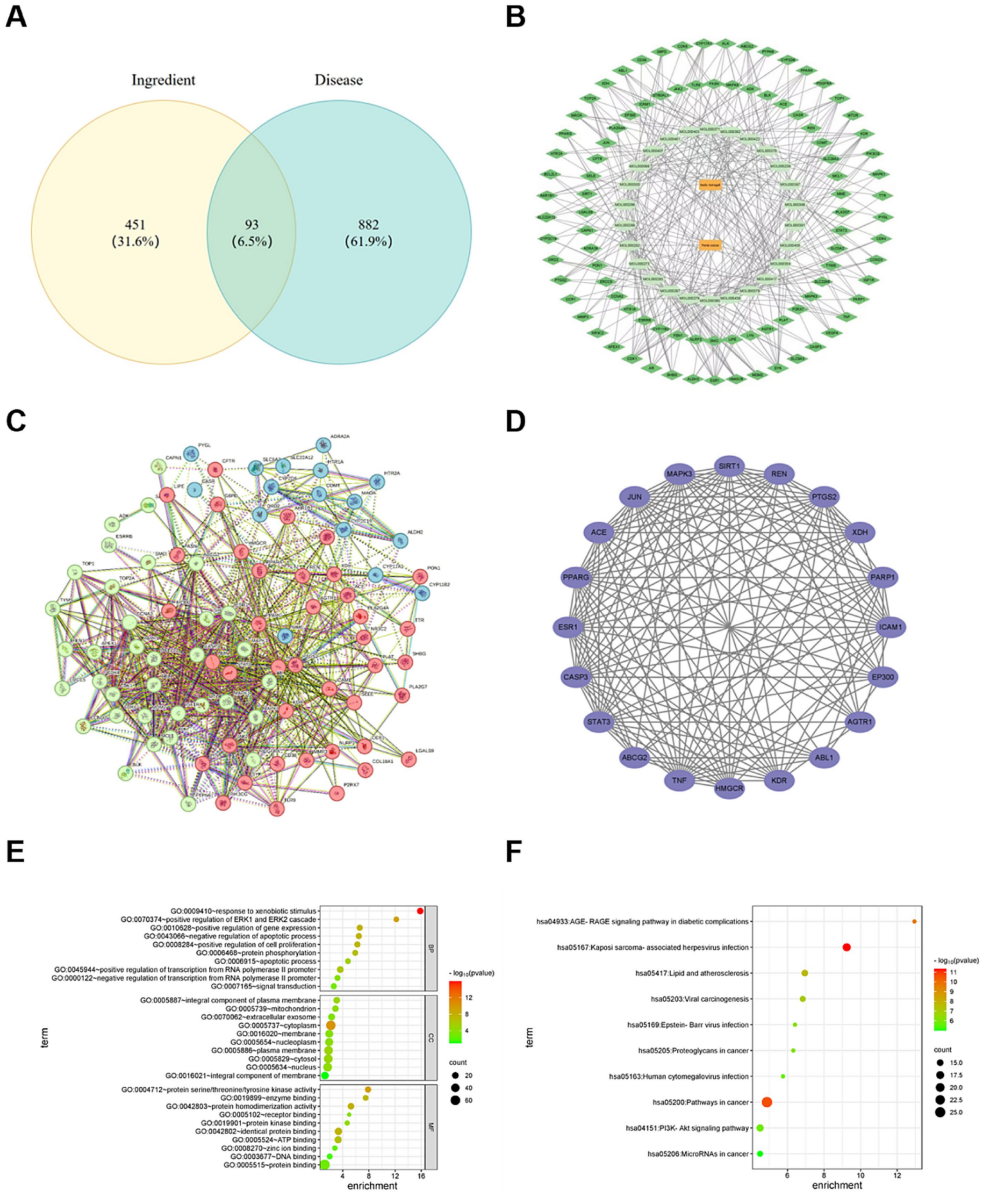
**(A)** Veen intersection targets diagram of RA, *Poria cocos* and HUA. **(B)** Diagram of the interaction network of the “drug component target” of RA and *Poria cocos* in the treatment of HUA. **(C)** Protein–protein interaction network of RA and *Poria cocos* in the treatment of HUA. **(D)** Core Targets. **(E)** GO function enrichment bubble. **(F)** KEGG pathway enrichment bubble.

The 975 HUA targets were retrieved from the GeneCards, DisGeNET, and MOIM databases. There were 93 common targets were found between HUA and the active compounds of RA and *Poria cocos*, visualized in a Venn diagram in Figure 5A.

#### 3.5.3 Construction of drug component target interaction network

The components and target data were introduced into Cytoscape3.9.1 to obtain the interactive effect network diagram representing “drug component target.” In Figure 5B, the orange rectangles represent drugs, deep green rhombuses represent targets and aqua ovals represent the components.

#### 3.5.4 Construction of PPI network

To construct the PPI network, 875 edges were sourced from the STRING database and the consequence was performed in Figure 5C. The core targets, selected based on degree values, betweenness centrality and closeness centrality, are shown in Figure 5D. The top five degrees values of core targets were TNF, STAT3, CASP3, JUN, and ESR1, showing eminent regulatory potential in HUA treatment. TNF, especially TNF-*α*, a pro-inflammatory cytokine, plays an important role in the pathological progression of HUA. Previous study revealed that UA could increase the level of TNF-α and stimulate the release of IL-1β (55). STAT3 plays a significant role in many cellular processes, such as cell growth and cell apoptosis (56). It exerts an important influence on the process of renal fibrosis. Inhibiting STAT3 expression could alleviate the kidney disease caused by HUA (57). CASP3 play a key role in the process of cell apoptosis (58). The decrease in CASP3 expression could improve renal tubule dilation and degeneration of epithelial cells (59). JUN is involved in the growth regulation of tumor cells (60). Some studies showed that high level of UA is associated with a high incidence of cancer (61, 62). The abnormal expression of JUN may lead to the increase in UA level. ESR1 can mediate estrogen signals in the body and may exert an influence on PI3K/AKT signaling pathway. Therefore, the results show that RA and *Poria cocos* play therapeutic role in HUA via multiple target points. The specific mechanism of action needs a further study.

#### 3.5.5 Consequence of GO function analysis

Through GO enrichment pathway analysis, a total of 596 GO terms were obtained, including 457 BP, 50 items of CC, and 98 MF. In terms of BP, the items were involved in the regulation of transcription from RNA polymerase II promoter, the reaction to exogenous stimulus, the positive regulation of genetic expression, and so on. In terms of CC, the items were mainly involved in the plasma membrane, cytoplasm, and nucleus, etc. In terms of MF, the items were mainly related to protein binding, protein binding rate and ATP binding. The top 10 most significant terms of BP, CC and MF are shown, respectively, in Figure 5E. RA and *Poria cocos* play roles in HUA treatment by regulating various biological processes.

#### 3.5.6 Consequence of KEGG pathway analysis

A total of 681 signal pathways attained from KEGG enrichment pathway analysis, consisting of pathways in cancer, the PI3K-Akt signaling pathway, lipids and atherosclerosis, the AGEs-RAGE signaling pathways in diabetic complications, proteoglycans in cancer, and so on. The top 10 important terms are displayed in Figure 5F. A study has proven that AGEs can induce oxidative stress and inflammation in various cells and organs via combining with AGEs receptors (RAGE) (63). HMGB1 is significant mediator of inflammation and a high affinity ligand of RAGE (64). High concentrations of UA strikingly increased extracellular release of HMGB1 (65), promoting the release of IL-6 and TNF-*α* and other inflammatory cytokines, which lead to renal tubulointerstitial fibrosis and glomerular injury. UA can stimulate the expression of IL-1β, TNF-α, and PTGS2 through PI3K-Akt signaling pathway to induce inflammatory reaction of renal tubular epithelial cells. Inhibiting PI3K-Akt signaling pathway not only can decrease the expression of URAT1 and GLUT9, but also can alleviate kidney inflammatory injuries (66, 67). Therefore, it can be speculated that the treatment of HUA with RA through regulating in multiple signal pathways.

#### 3.5.7 Consequence of molecular docking

**Figure 6:**
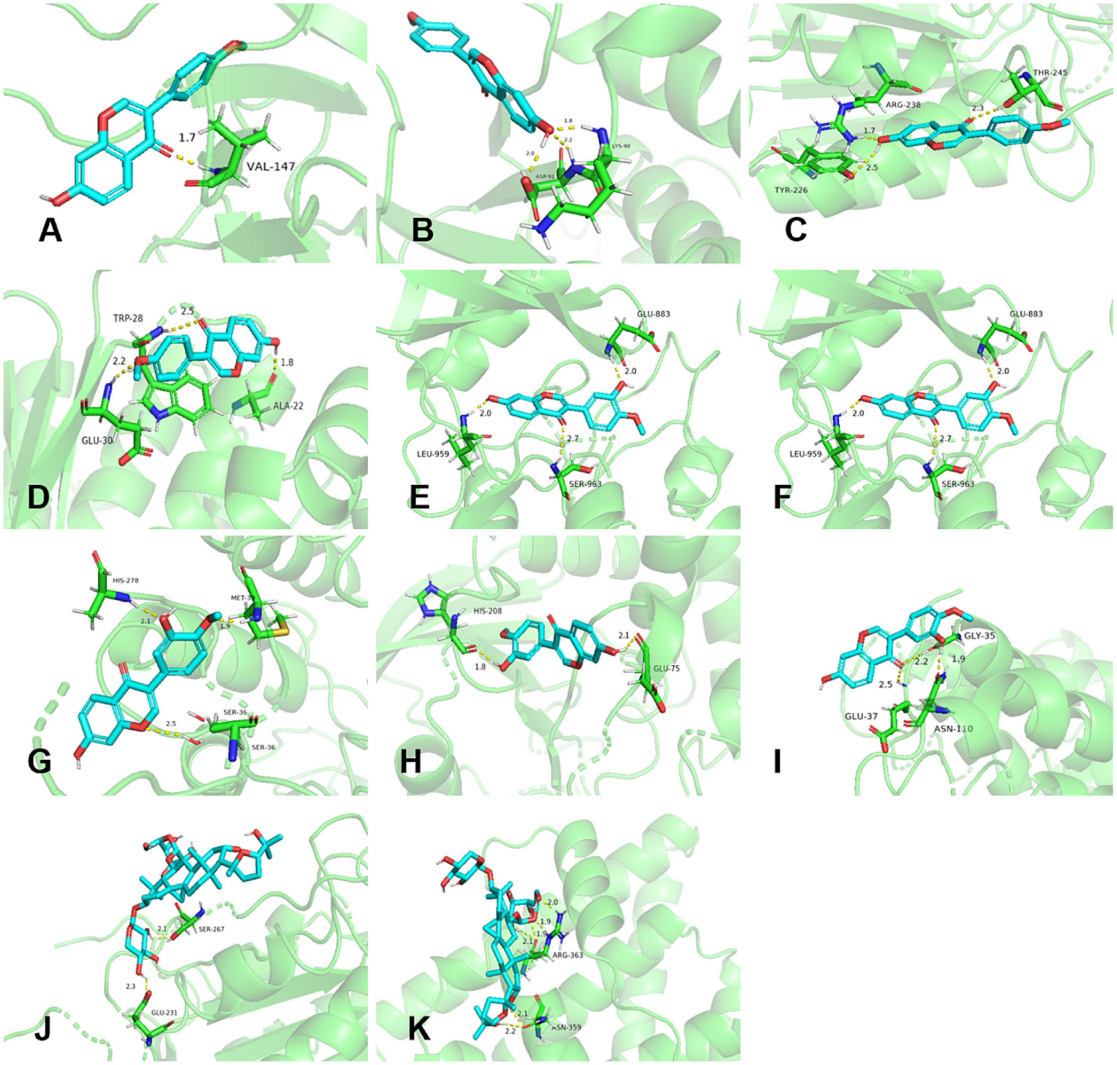
Molecular docking results of active components in RA and *Poria cocos* with core targets. **(A–E)** Formononetin with TNF, STAT3, CASP3, JUN, and ESR1. **(F–I)** Calycosin with STAT3, CASP3, JUN, and ESR1. **(J–K)** AG IV with CASP3 and ESR1.

Molecular docking consequence was evaluated based on affinity and was considered as strong interaction when affinity < −5.0 kcal/mol (68–71). The top five degrees values of the core targets performed molecular docking with AG IV, calycosin, and formononetin. Figure 6 shows that calycosin and formononetin bound well to TNF, STAT3, CASP3, JUN, and ESR1, with binding energies below −5.0 kcal/mol. AG IV bound well to CASP3 and ESR1, with binding energies below −3.0 kcal/mol.

## 4 Conclusion

In the current study, the results indicated that the fecal microbiota had a function on the metabolism of effective compounds in RA and FRA. After the *in vitro* fecal microbiota colonic fermentation, both FRA and RA displayed decreasing trends in contents of AG IV, total saponins, and flavonoids. In FRA group, the contents of AG IV, total saponins, and flavonoids were 0.60 ± 0.10 mg/g, 54.00 ± 2.11 mg/g, and 11.54 ± 1.33 mg/g at 48 h, respectively. While were 0.47 ± 0.04 mg/g, 43.31 ± 3.55 mg/g, and 7.04 ± 0.05 mg/g at 48 h in RA, respectively. The pH values of the FRA and RA decreased in the first 6 h and rose slightly in FRA after 6 h, while remained stable trend in RA after 6 h. The pH values decreased to 5.06 ± 0.00 and 4.66 ± 0.02 at 6 h in FRA and RA, respectively. In antioxidant assay, the antioxidant capacity of FRA was superior to RA in both DPPH and ABTS radical scavenging assessments at 0 h and 48 h. Furthermore, we found that *Lactobacillus acidophilus* displayed more conspicuous effects on the metabolism of effective compounds in contents of FRA, AG IV, total saponins, and flavonoids rose and then went down during the fermentation process. The contents of AG IV and total saponins were 1.14 ± 0.20 and 136.34 ± 6.15 mg/g at 12 h, respectively. The total content of flavonoids was 6.35 ± 0.06 mg/g at 6 h. In RA, the contents of AG IV and total saponins increased and then reduced in the fermentation course, peaking of 0.63 ± 0.05 mg/g at 12 h and 115.12 ± 4.12 mg/g at 24 h, respectively. The content of total flavonoids consecutively decreased during the fermentation course and decreased to 1.91 ± 0.18 mg/g at 48 h. The pH values in two groups displayed an uprising trend, following decrease. At the initial stage, the pH values of the FRA and RA groups were 6.21 ± 0.02 and 6.27 ± 0.01, respectively. At 6 h of fermentation, the pH values of the FRA and RA groups reached the highest level 6.86 ± 0.10 and 6.82 ± 0.42, respectively. Moreover, FRA group showed higher viable cell counts and better support in the growth of *Lactobacillus acidophilus* than RA.

The further pharmacodynamic experiment confirmed that the FRA did have more conspicuous functions in lowering UA levels, inhibiting XOD activity, decreasing TG, ALT, and AST levels in the HUA model than RA, showing better liver and kidney protective effects. The network pharmacology suggested that the functions of RA had the characteristics of multiple components, multiple target points, and multiple signal pathways in the treatment of HUA. The main active compounds in RA were likely to be AG IV, calycosin, and formononetin, the targets TNF, STAT3, CASP3, JUN, and ESR1 may be the core targets in the treatment of HUA, and the main signaling pathways were likely to be AGEs-RAGE pathway and PI3K/Akt pathway. Compared with current mainstream drugs in HUA treatment, FRA may show a safer and more hypotoxic in clinical treatment. This study reveals the mechanism of RA and *Poria cocos* in the treatment of HUA and may provide a scientific foundation for clinical utilization of FRA in the treatment of HUA. On the basis of this research, future studies will be necessary to clarify the mechanism of FRA on XOD level, UA reduction via interfering on intestinal flora and its metabolites and pharmacological mechanisms.

## Data Availability

The datasets presented in this study can be found in online repositories. The names of the repository/repositories and accession number(s) can be found in the article/supplementary material.

## Footnotes

^1^ http://tcmspw.com/index.php

^2^ http://www.swisstargetprediction.ch/

^3^ https://www.genecards.org/

^4^ http://www.disgenet.org/

^5^ http://www.Omim.org/

^6^ https://www.string-db.org/

^7^ https://www.rcsb.org/

^8^ https://pubchem.ncbi.nlm.nih.gov/
